# Facebook as a tool to promote radiology education: expanding from a local community of medical students to all of South America

**DOI:** 10.1590/0100-3984.2017.0112

**Published:** 2018

**Authors:** Matheus Zanon, Stephan Altmayer, Gabriel Sartori Pacini, Álvaro Guedes, Guilherme Watte, Edson Marchiori, Bruno Hochhegger

**Affiliations:** 1 Department of Diagnostic Methods, Universidade Federal de Ciências da Saúde de Porto Alegre (UFCSPA), Laboratório de Pesquisa em Imagens Médicas (Labimed), Porto Alegre, RS, Brazil.; 2 Department of Radiology, Universidade Federal do Rio de Janeiro (UFRJ), Rio de Janeiro, RJ, Brazil.

**Keywords:** Education, medical, continuing, Clinical medicine/education, Radiology/education, Social media

## Abstract

**Objective:**

To assess the feasibility of Facebook to promote a radiology education project and to expand it from our university community of medical students to a wider audience.

**Materials and Methods:**

A group of 12 medical students created a Facebook page in June 2015, to contribute to radiology education in our university. From August 2015, clinical cases, including a brief explanation of clinical findings, along with different imaging modalities, were posted weekly and subscribers were encouraged to choose the most appropriate diagnosis. All cases were followed by the appropriate answer and an explanation to highlight imaging findings and diagnosis. Aiming to reach a larger audience, we also shared cases to a public Latin-American Facebook group, comprising a collective total of 28,182 physicians and medical students. Using the Facebook Insights tracking tool, we prospectively analyzed subscriber interaction with our page for 14 months.

**Results:**

During the period analyzed, 35 cases were posted. The most common imaging modalities were X-ray (n = 15) and computed tomography (n = 13). Before we began posting the weekly cases, our page had 286 likes. By October 2016, that number had grown to 4244, corresponding to an increase of 1484% and eight times the size of the medical student community at our institution (n = 530). Medical students made up most (76%) of the subscribers, followed by radiology residents (6%). An excellent or moderate contribution to personal image interpretation skills was reported by 65.3% and 33.1% of the users, respectively.

**Conclusion:**

Creating a Facebook page and posting weekly clinical cases proved to be an effective method of promoting radiology education.

## INTRODUCTION

Radiology is still not given sufficient attention during medical education. Only 25% of medical schools in the United States require radiology as a clinical rotation^(1)^. In the United Kingdom, only 5% of total teaching time in medical education is committed to radiology, which is not sufficient for students to be properly prepared for medical practice^(2)^. At many medical schools, one alternative for medical students to become more familiar with the radiology is to affiliate themselves with one of the interest groups-also known as "academic societies".

At the beginning of 2015, we created an academic society for diagnostic imaging at our university, designating it the Liga de Diagnóstico por Imagem (LiDi, Diagnostic Imaging League). Our goal was to supplement education in radiology and image interpretation for medical students at our institution. Once a week, meetings were held to discuss selected teaching topics and a medical student presented a clinical case, with the assistance of a senior radiologist responsible for our interest group. Any medical student enrolled at our university could attend the meetings. Because there was an increasing demand for students engaged in these activities, we tried to find ways to reach more people and expand the number of participants.

Facebook is the most popular social network worldwide, with 1.71 billion monthly active users in the second quarter of 2016^(3)^. As recent studies suggest^(4-10)^, Facebook has been recognized by medical institutions and professionals as a means of promoting research projects, delivering health information, and facilitating student education. Therefore, we decided to use Facebook as a tool to disseminate the clinical cases presented in our weekly meetings and to reach out a larger number of participants outside of our local student community.

The aim of this study was to assess the feasibility of Facebook as a tool to promote a local radiology education project and to expand that local project to a larger audience. An additional objective was to analyze the demographic characteristics and interests of the users of the Facebook page created.

## MATERIALS AND METHODS

### Clinical case design

**Figure 1:**
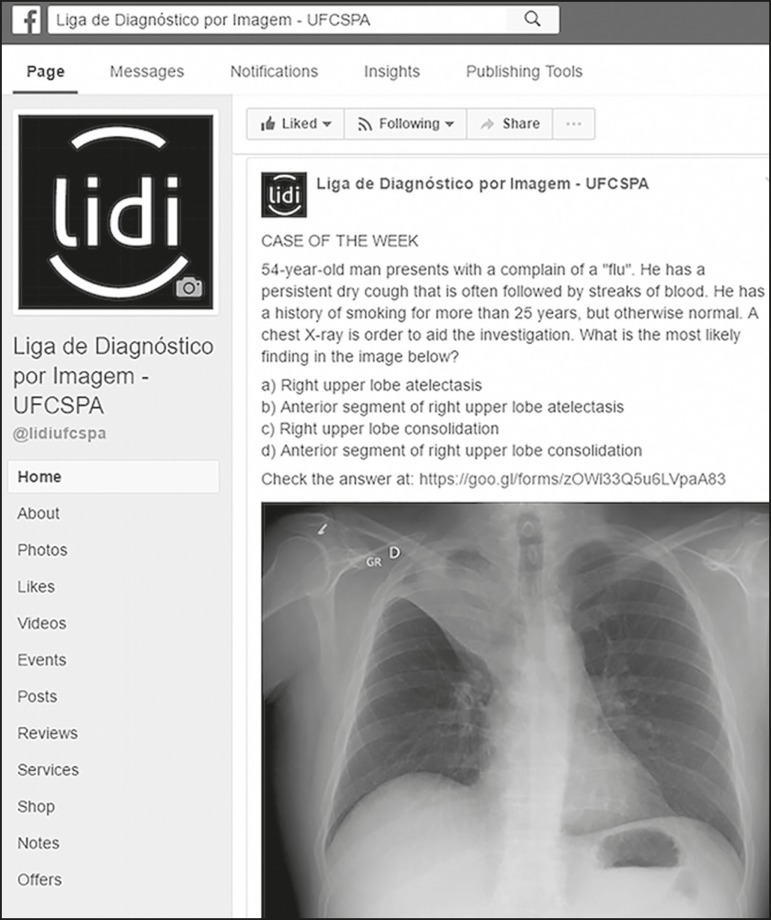
Example of one of the clinical cases posted on our page. The original data was posted in Portuguese. For this case, there were 1637 clicks on the post; the post reached 4764 people; and 103 users "liked", "commented on", or "shared" the content.

The LiDi Facebook page (www.facebook.com/lidiufcspa) was created on June 22, 2015, as a nonprofit page, administrated by 12 medical students. A series of clinical cases have been posted on the page, one a week since August 24, 2015. The posts consisted of a brief patient history and physical examination findings, along with different imaging modalities relevant to the case, such as X-ray, computed tomography (CT), magnetic resonance imaging (MRI), and ultrasound. Five possible diagnoses were provided, and the public was encouraged to choose the most likely diagnosis based on the vignette and images provided. For each case, the correct answer was followed by an explanation of the disease and commentaries to highlight the most important imaging findings. Figure 1 depicts an example of the standards followed for case posting.

Most topics and imaging modalities were chosen on the basis of the curriculum framework for undergraduate education in radiology proposed by the European Society of Radiology (ESR)^(11)^. We set an easy to moderate level of difficulty, as rated by the senior radiologist. In addition to the clinical cases, one extra weekly post per week focused on radiology teaching topics geared to medical students and residents was also planned.

To reach a larger audience, the medical student responsible for preparing the case of the week also shared the images to a public Latin-American Facebook group with 28,182 participants, composed mainly of physicians and medical students, that was created with the goal of sharing scientific papers, reviews, and case reports. We did not solicit any publicity via Facebook to gain visibility, likes, or number of subscribers.

### Data analysis

From August 2015 to October 2016, we prospectively analyzed user feedback on the content posted on our page. Public response was assessed using Facebook Audience Insights, a tracking tool that provides data about user demographics, engagement, likes, top posts, and number of visits. A "like" establishes a connection between a user and a Facebook page, the user thereafter receiving notifications about all the information posted, including photos, videos, links, status updates, and polls^(3)^. Other measures of user feedback about a page are "reach" and "engagement". The former indicates the number of people who have visualized any content, not necessarily interacting with the post, whereas the latter is a more precise measure of user reactions to the content, analyzing the number of likes, views, and shares^(3)^.

Because of Facebook's privacy policies, it is not possible to collect any user data other than sex, age, language, and location. Therefore, using Google Forms, we created a questionnaire that we shared on our Facebook page and encouraged users to complete. We thus collected data regarding the occupation of the users, as well as their opinions on the quality of the cases, topics, and imaging modalities they would like to see addressed. The questionnaire was live (i.e., responses were accepted) for a month.

### Statistical analysis

Statistical analysis was performed with the IBM SPSS Statistics software package, version 21.0 (IBM Corporation; Armonk, NY, USA). Data analysis included descriptive statistics, and variables are reported as mean ± standard deviation, range, or relative frequency, as appropriate. In addition, we calculated the number of views proportional to that of engaged users over time. That was done to evaluate whether any growth in the number of users was accompanied by a proportional increase in the interactions with the content. Group comparisons were made using one-way analysis of variance. For all statistical analyses, the level of significance was set at *p* ≤ 0.05. Due to the nature of this study, institutional review board approval was not required.

## RESULTS

**Figure 2:**
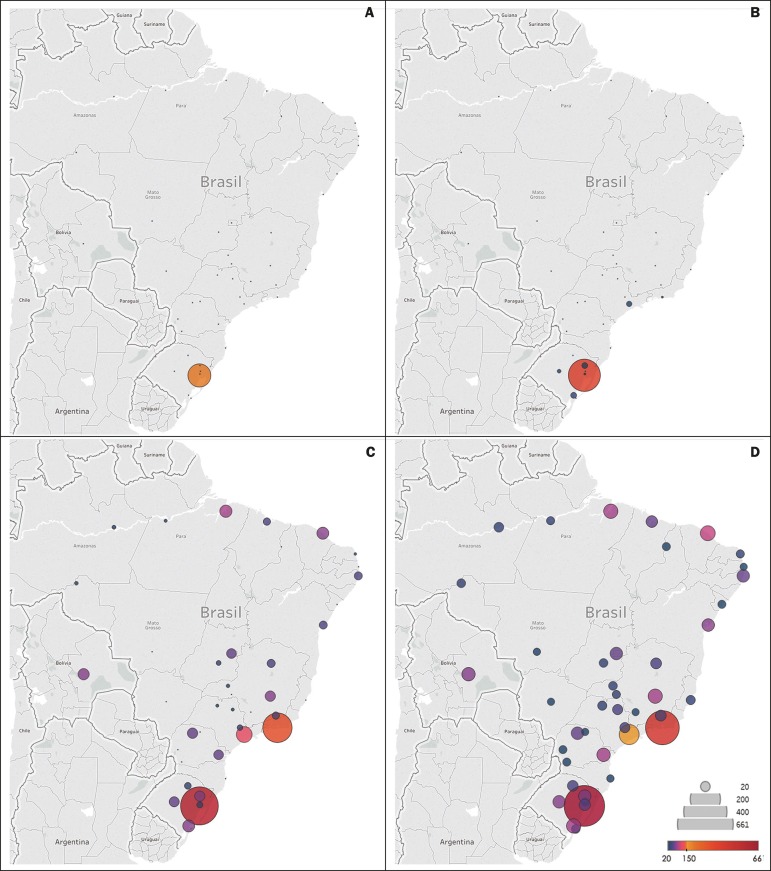
Quantitative maps showing the geographic distribution of subscribers to the LiDi Facebook page over time. Only cities with more than 20 users are represented. Initially, subscribers were concentrated in Porto Alegre, where our university is located. During the period analyzed, the number of users increased and the reach extended to the rest of Brazil, as well as to other countries, such as Bolivia. **A:** baseline; **B:** 0-140 days; **C:** 140-280 days; **D:** 280-420 days.

Thirty-five cases were posted in the period. As shown in Table 1, X-ray (n = 15) and CT (n = 13) were the most common imaging methods, chest X-ray being the topic most often addressed (n = 9). Before we began posting the clinical cases, our page had 286 likes. By October 2016, that number had risen to 4244, representing an increase of approximately 1484% and eight times the size of the medical student community at our institution (n = 530). The demographic characteristics of the subscribers are also shown in Table 1. Most were 18-24 years of age (n = 2037; 48%) or 25-34 years of age (n = 1867; 44%), and 58% (n = 2461) were women. Most of the users (n = 3919; 90%) accessed the page via a Desktop computer. Initially, the users were concentrated in Porto Alegre, where our university is located. During the period analyzed, the number of users increased and their distribution expanded to all of Brazil, as well as to other countries, such as Bolivia. Figure 2 provides a quantitative depiction of the geographic distribution of subscribers to the LiDi Facebook page over time. Users in Brazil accounted for 93% of the subscribers (n = 3943), whereas those in other South American countries (Argentina, Bolivia, Ecuador, Peru, Uruguay, and Venezuela) collectively accounted for 3% (n = 121). The other 4% were mostly in the European Union or United States.

**Table 1:** Case characteristics and page subscriber demographics.

Parameter	N	%	Variation (%)
Imaging modality			
X-ray	15	45.45	-
CT	12	36.36	-
MRI	3	9.09	-
Mammography	3	9.09	-
Ultrasonography	2	6.06	-
Topic			
Chest	9	27.27	-
Abdomen	7	21.21	-
Musculoskeletal	8	24.24	-
Central nervous system	5	15.15	-
Gynecology & Obstetrics	4	12.12	-
Number of likes			
Baseline	286		-
0-140 days	888		310.49
140-280 days	3328		374.77
280-420 days	4244		127.52
Overall	3958		1483.92
Female	2461	58	-
Age range			
18-24 years	2037	48	-
25-34 years	1867	44	-
35-44 years	212	5	-
Other	128	3	-
Location			
Brazil	3943	93	-
Latin-America	121	3	-
Other	180	4	-
Language			
Portuguese	3682	86	-
English	313	7	-
Spanish	99	2	-
Other	150	5	-

-, Not applicable.

**Figure 3:**
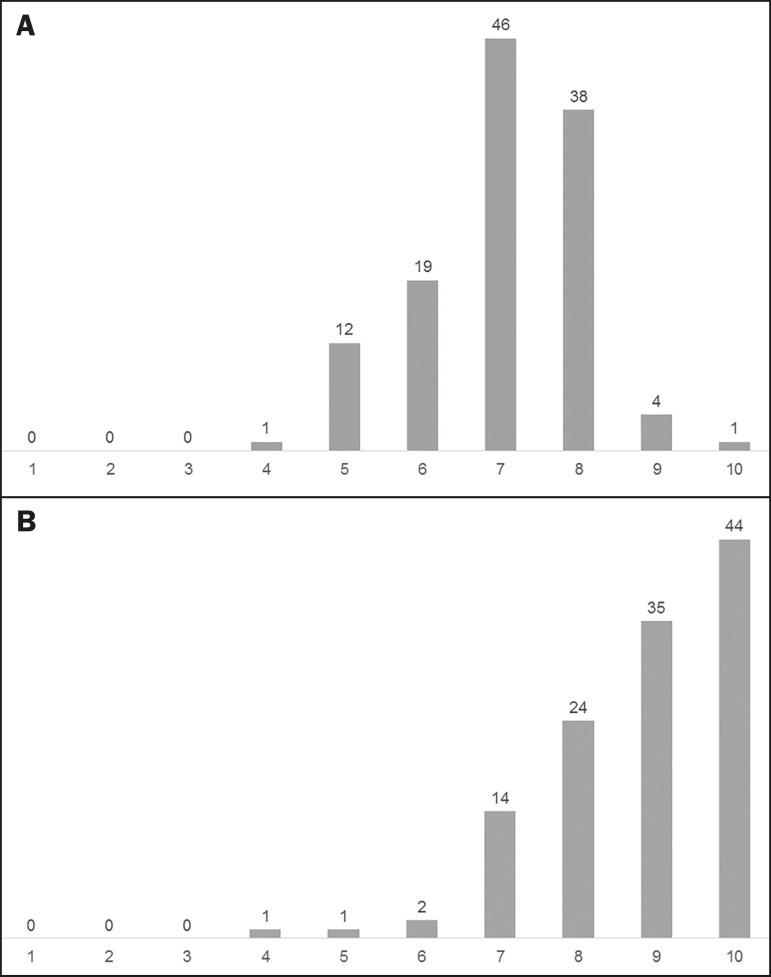
User evaluations (n = 121) of the level of difficulty and quality of the clinical cases (**A** and **B**, respectively), on a scale of one to ten.

The Google Forms questionnaire comprised 121 questions designed to assess user opinions and characteristics (Table 2). Undergraduate medical students made up the majority of the respondents (76%), followed by radiology residents (5.8%). Figure 3 presents user opinions about the quality and level of difficulty of the cases. When the respondents were asked what they would like to see featured in future cases, the most commonly requested topics were neuroradiology (n = 73; 60.3%), chest X-ray (n = 68; 56.2%), and abdominal X-ray (n = 63; 52.1%), whereas the most commonly requested imaging modalities were CT (n = 97; 80.2%), MRI (n = 90; 74.4%), X-ray (n = 70; 57.9%), and ultrasound (n = 61; 50.4%). Most (n = 76; 62.8%) of the users reported that they had read the explanation provided along with the cases through Google Docs. However, 34 (28.1%) did not even know that option existed. An excellent or moderate contribution to personal image interpretation skills was reported by 79 (65.3%) and 40 (33.1%) of the users, respectively. Only two users (1.7%) reported that the contribution was slight, and none of the users reported that there was no contribution.

**Table 2:** Subscriber occupations.

Occupation	N	%
Undergraduate students		
Medicine	92	76.0
Physiotherapy	3	2.5
Other	3	2.5
Residents		
Radiology	7	5.8
Internal medicine	1	0.8
Other	5	4.1
Consultant		
Radiology	1	0.8
Other	7	5.8
Other health professional	2	1.6

*Note*: Data were collected with a Google Forms questionnaire.

**Figure 4:**
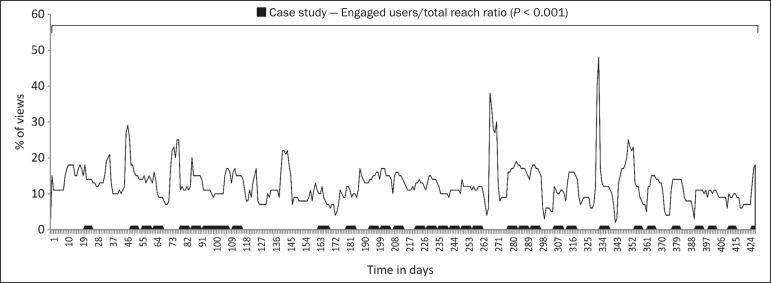
Distribution of engaged users per total reach ratio over time. There was a statistically significant increase in user engagement over the period analyzed (*p* < 0.001), and the peaks corresponded to the dates when clinical cases were posted.

Analysis of the engaged users per total reach ratio during the period assessed demonstrated a significant increase in subscriber interaction over time (*p* < 0.001). It is noteworthy that the peaks in interaction occurred on the dates when clinical cases were posted on the page (Figure 4).

## DISCUSSION

We found that Facebook was a valuable tool to promote our radiology education project and to compensate for the potential lack of undergraduate training in the subject, because most users reported a significant positive impact on their personal image interpretation skills. In addition, the weekly posting of a clinical case with a multiple-choice question was found to improve the LiDi page's growth rate and subscriber interaction with the posts.

Other authors have recognized the utility of Facebook as a tool to promote research projects, deliver health information, and facilitate medical education^(3-5)^. Social media other than Facebook (e.g., Twitter, Instagram, and YouTube) have also contributed to the dissemination of medical education^(6,7)^. Because they are free resources, social media facilitate interactivity and information sharing. For instance, Hoang et al.^(8)^ demonstrated that a specific educational point is viewed six times more frequently in blogs, such as Radiopedia.org (a collaborative blog on Radiology), than in scientific papers from the American Journal of Roentgenology or the American Journal of Neuroradiology.

In our experience, X-rays were the most common type of examination addressed in the clinical cases. As a common first-line imaging modality, we believed that this would be the method of greatest interest for undergraduate medical students. According to a publication by the ESR on undergraduate education in radiology, students should display a systematic approach to the comprehensive interpretation of X-rays, being able to detect abnormalities on X-rays of the chest, abdomen, and skeleton, as well as being able to relate the findings to clinical management^(11)^. Although there was a slightly superior prevalence of cases focusing on chest conditions (n = 9), we tried to maintain an equal distribution of topics related to conditions affecting the abdomen (n = 7) and skeleton (n = 8), the same areas recommended by the ESR guidelines. Other imaging modalities, such as MRI and mammography, and other areas of study, such as neuroradiology and gynecology/obstetrics, were also discussed, although they were given less emphasis.

Our LiDi Facebook page also provided other content, such as educational videos/lessons, curiosities about glossary terms in radiology, and information about the latest advances in imaging technology. However, those posts garnered less engagement of the public than did the clinical cases, in accordance with the findings of a previous study^(3)^.

One limitation of our study is that student performance after exposure to the content was not assessed. Initiatives disposed to create similar Facebook pages should try to use free online platforms, such as Google Forms, in order to monitor user performance prospectively. Use of Facebook compared with other learning and teaching environments or in comparison with different social media tools should also be investigated.

We demonstrated that radiology education could also benefit from the use of social media. Posting cases weekly helped attract more users and engage subscribers with the content. We conclude that creating a Facebook page based on weekly clinical cases can be a valuable method to promote radiology training.
